# Investigation of the Effects of Purpose in Life, Grit, Gratitude, and School Belonging on Mental Distress among Chinese Emerging Adults

**DOI:** 10.3390/ijerph15102147

**Published:** 2018-09-29

**Authors:** Meng Xuan Zhang, Ngai Lam Mou, Kwok Kit Tong, Anise M. S. Wu

**Affiliations:** Department of Psychology, Faculty of Social Sciences, University of Macau, Avenida da Universidade, Taipa, Macao, China; yb77304@connect.umac.mo (M.X.Z.); sb61952@connect.umac.mo (N.L.M.); kktong@umac.mo (K.K.T.)

**Keywords:** purpose in life, gratitude, grit, school belonging, depression, anxiety, stress, emerging adults, Chinese

## Abstract

Given the high prevalence of mental distress indicators, such as depression, among emerging adults, it is imperative to identify not only factors that place them at risk for mental distress, but also those that protect against it. This study tested the direct and indirect effects (via purpose in life) of gratitude, two aspects of grit (i.e., consistency of interest and perseverance of effort), and school belonging on three indicators of mental distress (i.e., depression, anxiety, and stress). A total of 468 Chinese university students (58.3% female), aged 18 to 27, in Macao, China responded to an anonymous questionnaire between April to May, 2016. As expected, all psychosocial factors were negatively associated with all three indicators of mental distress (*r* = −0.15 to −0.42, *p* < 0.05), with the exception of perseverance of effort, which had a significant, negative association with depression only. The results of path analysis showed that purpose in life significantly mediated the effect of school belonging and perseverance of effort on depression, whereas school belonging, gratitude, and consistency of interest all had direct effects on all three indicators of mental distress (*p* < 0.05). Our results also suggested that the two components of grit may have differential effects on mental distress among Chinese emerging adults. School-based programs should consider positive psychology interventions in Chinese populations.

## 1. Introduction

During the transition to adulthood (i.e., emerging adulthood), multiple developmental tasks, including educational pursuits, post-graduation life planning, and personal relationships, make people more vulnerable to mental health problems, including depression and anxiety disorders [1]. For example, in a national survey, Australian adults between the ages of 18 and 24 reported the highest prevalence (27%) of any mental disorder in the past year compared to all other age groups [2]. University students in this transitional period have shown a similar, or an even higher, prevalence rate of mental distress [1,3]. According to a review of 24 studies published between 1990 and 2010, the weighted mean prevalence of probable depression among university students was 31%, which was higher than the 9% reported in the general population [1,3]. Among American university students, 46% had a psychiatric disorder in the past year, with 11% reporting mood disorders and 12% anxiety disorders [4], while the prevalence of moderate or above levels of depression, anxiety, and stress was as high as 21%, 41%, and 27%, respectively, in Hong Kong (China) [5]. There is also evidence that suicide attempts reach the peak during adolescence and emerging adulthood (i.e., ages of 15–24 years) [6]. Among Chinese university students, approximately 11% of the pooled prevalence of suicidal ideation was reported in a meta-analysis study [7]. Thus, the mental health of Chinese emerging adults calls for more research and clinical attention, and preventive interventions for mental distress and related disorders, such as depression and anxiety, in emerging adulthood is warranted.

In comparison to later-onset mental illness or the absence of mental health, mental disorders during or before emerging adulthood are likely to have a negative impact on educational attainment, resulting in lower functioning in civic life and lower future earnings [8,9]. To date, prevention efforts have relied primarily on risk factors for university students’ mental health, such as childhood trauma, relationship stressors, sexual identity, and financial difficulties [10,11,12,13]. However, rather limited knowledge has been gleaned on protective psychological factors, particularly in Chinese populations. Therefore, identifying such factors among emerging adults is imperative.

### 1.1. The Protective Effect of Purpose in Life in Emerging Adulthoods

According to Arnett, identity formation is a key developmental task during emerging adulthood [14]. Bronk argued that developing a sense of purpose, defined as an enduring and meaningful commitment to a task or a desire one wants to accomplish in life, will facilitate identify formation, which will, in turn, strengthen commitment to one’s purpose [15]. In other words, it appears that identifying one’s purpose in life is helpful to youth’s ability to navigate this developmental task adequately [16]. In a Dutch national panel study that interviewed youth between the ages of 15 and 24 in two waves, relational- and vocational-identify formation were found to be associated with better mental health [17]. Similarly, Brassai et al. found that adolescents with higher levels of meaning in life reported fewer health-related behavior problems, such as drug use, lack of exercise, and unsafe sex; they argued that having meaning in life is related to lower psychosomatic symptoms and appears to serve a protective role [18]. Young adults who report having a purpose in life also report higher levels of hope and lower levels of suicidal ideation and problematic gaming than those reporting lower levels of purpose in life [19,20,21]. Therefore, a sense of purpose in life (i.e., an intention to exert commitment and goal-directedness toward a goal that is personal, meaningful, and which at times is beyond-the-self) is believed to be a salient protective factor against poor mental health among Chinese emerging adults. Although there is evidence that suggests that purpose in life contributes to positive wellbeing (e.g., positive affect and life satisfaction), and is a protective factor in the process of acculturation, among Chinese students in Australia [22,23,24], its impact on negative mental health outcomes (e.g., depression and anxiety) among Chinese emerging adults is unclear. The current study aims to bridging this research gap.

Although the development of purpose in life can start in childhood, individuals do not consider it seriously until they arrive at the transition to adulthood [16]. In addition to its direct protective effect, purpose in life may also protect an emerging adult’s wellbeing by transforming other positive character traits, such as grit and gratitude, into resiliency against mental distress and problems (e.g., suicidal ideation) [20]. By definition, both grit and gratitude serve as resiliency factors, but grit is regarded as an intrapersonal strength, whereas gratitude is an interpersonal one [25]. Grit and gratitude are generally expected to enhance the strength and awareness of one’s purpose in life, which are proposed to be salient determinants of his/her resource allocation and behavioral flexibility, and in turn wellbeing [26]. The mediating effect of purpose in life on the relationship between positive character traits and subjective wellbeing was documented [27]. Further research is warranted to test whether the findings are replicable in other emerging adult populations.

### 1.2. Protective Roles of Grit and Gratitude

Grit is defined as the strength to maintain effort and interest toward long-term goals [28]. There are two major components in grit: consistency of interests, which is the ability to maintain a similar array of interests over years, and perseverance of effort, which is the ability to sustain one’s efforts in spite of adversity [28]. Thus, grit is future-oriented, and it strengthens one’s ability to endure and commit to meaningful goals, such as purpose in life [28,29]. Indeed, evidence showed that individuals with more grit exhibit a stronger sense of purpose. The findings of Kleiman and his colleagues also suggested that university students who possess the ability to be content with the good and to persevere through the bad are likely to develop a stronger sense of meaningfulness in their lives [20]. University students in Canada and the United States who demonstrated stronger grit also displayed stronger positive affect and purpose commitment [30].

Positive correlations between grit and subjective wellbeing have been well documented [20,31,32,33]. For example, grit was shown to have positive correlations with positive affect and happiness [34], whereas it had negative correlations with negative affect, stress, and depression [34,35]. However, one should note that the two components of grit (i.e., consistency of interest and perseverance of effort) may not always exert the same effect on subjective wellbeing [36]. For instance, in a Filipino university student sample, only perseverance of effort improved life satisfaction and positive affect, while consistency of interests had no significant effect at all [31]. Compared to consistency of interest, perseverance of effort was also found to have a stronger relationship with a mental state of complete absorption and full mastery in the preferred activities [37]. However, there is a lack of knowledge regarding whether components of grit have differential effects on mental health problems.

Gratitude is “a generalized tendency to recognize and respond with grateful emotion to the roles of other people’s benevolence in the positive experiences and outcomes that one obtains” [38] (p. 112). This positive personal character attribute has been shown to be a protective factor against mental distress. In a review by Wood et al. [39], gratitude was shown to have negative associations with psychopathological conditions, such as depression, generalized anxiety disorder, substance dependence, and bulimia nervosa, whereas, appreciation for the value and contributions of others seems to be a shared characteristic of youth with a sense of purpose in life, a finding that has been observed in both quantitative and qualitative studies [20,40].

### 1.3. The Role of School Factor: School Belonging

A generalized sense of belonging has been found to be a predictor of meaning in life as well [41]. Since emerging adults spend a considerable amount of time in school for personal advancement or career preparation, it is conceivable that a stronger social connection to school may also enhance their purpose in life. Hence, school belonging, which is defined as “the extent to which students feel personally accepted, respected, included, and supported by others in the school social environment” [42] (p. 80), needs to be examined as a potential protective factor against mental distress among emerging adults.

School belonging has been found to be a salient correlate of students’ psychological well-being [43]. Low sense of school connectedness has also been identified as a predictor of depressive symptoms and anxiety symptoms among adolescents [44] and a significant correlate of internalizing problem behaviors among university students [45]. Among Chinese undergraduate students, school belonging was shown to have a positive relationship with gratitude [46], but the relationship between school belonging and either grit or purpose in life is not clear.

Despite the lack of empirical evidence for the association between school belonging and purpose in life, some indicators of the former have been found to be positively correlated with the latter among students. For example, perceived mentor support, which is often involved in school belonging formation, was identified as a key element in youth’s development of purpose [47,48]. Having a positive relationship with mentors (e.g., teachers) at school is believed to help adolescents and emerging adults discover and pursue their purposes [47]. Explicit and well-established school values, school policies, curriculum, and extracurricular activities serve to satisfy students’ interest and developmental needs and can together create a positive culture to support school belonging [43]; it is within such positive cultures that university students’ purpose can be intentionally fostered [49]. Therefore, school belonging may facilitate a sense of purpose in life among those emerging adults who spend time in school settings.

### 1.4. The Present Study

Considerable cultural variations in the prevalence, associated factors, and outcomes of mental disorders have been observed [50,51,52], and research in local populations of a specific culture is called for [53]. The present study aimed to investigate whether purpose in life mediates the protective effects of three positive character traits on mental distress among Chinese university students. The study examined both the direct and indirect effects (via purpose of life) of the positive character traits of gratitude, grit (i.e., consistency of interest and perseverance of effort), and school belonging on mental distress in Chinese emerging adults. Based on the findings of previous studies, the following hypotheses were formulated and tested: (a) there is a negative correlation between consistency of interest, perseverance of effort, gratitude, school belonging as well as purpose in life and mental distress; (b) gratitude, consistency of interest, perseverance of effort, and school belonging have direct effects on depression, anxiety, and stress; (c) purpose in life mediates the effect of these factors on depression, anxiety and stress.

## 2. Materials and Methods

### 2.1. Participants and Procedures

From April to May 2016, Chinese students, aged 18 to 27 years (both genders: 1 = male; 2 = female), were recruited to join our survey, after their lectures of language courses in the general education program, which serves all students at a public university in Macao, China. A total of 468 Chinese adults, whose ages ranged from 18 to 27 years old (Mean age = 19.29, *SD* = 1.10; 58.3% female) voluntarily participated in the study, a majority (96.4%) of whom were in their first or second year of undergraduate study. Before filling out an anonymous questionnaire, they were clearly explained their rights, including the right to withdraw from participation at any time without penalty, and they provided their written consent to participate. The participants received a supermarket coupon of 100 MOP (about 12.5 USD) as a token of appreciation for participating. This study was conducted after obtaining an approval from the ethics committee of the affiliated university of the corresponding author (MYRG 2015-00213-FSS).

### 2.2. Measures

Mental distress was measured by the Depression Anxiety Stress Scale (DASS-21) [54]. The DASS-21 contains three 7-item subscales that assess depression (e.g., “I couldn’t seem to experience any positive feeling at all”), anxiety (e.g., “I was aware of dryness of my mouth”), and stress (e.g., “I found it hard to wind down”), respectively. Participants rated how often they experienced such symptoms over the past week on a 4-point Likert scale (in which 0 = “did not apply to me at all” and 3 = “applied to me very much”, or “most of the time”). Higher scores indicated higher levels of distress. Based on their subscale scores, the participants were also classified into four groups regarding each type of distress: normal (Depression: 0–9; Anxiety: 0–7; Stress: 0–14), mild (Depression: 10–13; Anxiety: 8–9; Stress: 15–18), moderate (Depression: 14–20; Anxiety: 10–14; Stress: 19–25) and severe or above (Depression: 21+; Anxiety; 15+; Stress: 26+) [54]. Cronbach’s alpha was 0.89, 0.86, and 0.86 for depression, anxiety, and stress subscales, respectively.

Purpose in life was assessed by the 6-item Purpose in Life Test (PIL) [55]. Each item was rated on a 5-point scale that was specifically designed for that item (e.g., “My life is ____, with scores ranging from 0 = ‘empty’ to 5 = ‘exciting’”). A higher score indicates a higher level of purpose in life. In this study, Cronbach’s alpha was 0.81.

*Grit* was measured by the 12-item Grit Scale [28], which considers the two facets of grit: Consistency of interests (Grit-C; e.g., “My interests change from year to year” [reverse scored]) and perseverance of effort (Grit-P; e.g., “I finish whatever I begin”). Each item is rated on a 5-point Likert scale, from 1 = “strongly disagree” to 5 = “strongly agree”. After recoding, a higher score indicated a higher level of grit. In this study, Cronbach’s alpha for consistency of interests and perseverance of effort was 0.77 and 0.78, respectively.

Gratitude was assessed by the Gratitude Questionnaire (GQ-6) [38], which is a 6-item measure that assesses one’s general grateful affect (e.g., “I am grateful to a wide variety of people”) on a 7-point Likert scale (in which 1 = “strongly disagree” and 7 = “strongly agree”). Higher scores represent higher levels of gratitude. In this study, Cronbach’s alpha for the scale was 0.80.

School belonging was assessed by the 18-item Psychological Sense of School Membership Scale (PSSM) [42], on a 6-point Likert scale, in which 1 = “strongly disagree” and 6 = “strongly agree”. A sample item is, “I felt like a real part of my school”. A higher score indicates a higher sense of school belonging. This Chinese version of the scale had a Cronbach’s alpha of 0.85.

### 2.3. Data Analyses 

SPSS 24 (IBM, Armonk, NY, USA) was used to conduct preliminary statistics, including examining the inter-correlations of all the variables. In order to test both direct and mediating effects of purpose in life, path analysis was conducted with AMOS 24 (IBM, Armonk, NY, USA) for examining the mediation models, in which purpose of life mediated the relationship between the three psychological variables (i.e., grit, gratitude, school belonging) and the three indicators of mental distress (i.e., depression, anxiety, and stress). The full mediation model was first tested and then modified according to the results of path coefficient tests and modification index tests. Goodness-of-fit statistics were used to assess the model. Good model fit was deemed to be demonstrated by the following fit indices: comparative fit index (CFI), which should exceed 0.9 [56], as well as root mean square error of approximation (RMSEA) and standardized root mean square residual (SRMR), which should be less than 0.08 [57]. Moreover, a nonsignificant chi-square would indicate a good model fit, and the relative chi-square (chi-square divided by degree of freedom) should not be more than 3 [58]. Moreover, standardized coefficients were estimated with 95% confidence interval based on the bias-corrected percentile method with 5000 bootstrap samples.

## 3. Results

### 3.1. Preliminary Statistics

Of the 468 participants, 11.7% participants reported mild anxiety, whereas 20.1% and 6.2% reported moderate and severe or above anxiety, respectively. For depression symptoms, 14.1% participants reported a mild level of depression, whereas 9.2% reported moderate depression, and 0.6% severe or above depression. Only 5.5% participants reported stress at the mild level or above.

### 3.2. Correlation Analysis

Gratitude, school belonging, and grit-C were negatively correlated with depression, anxiety, and stress (*p* < 0.05); however, grit-P had a significant, negative association with depression only. Purpose in life had a significant, negative association with stress (*r* = −0.23, *p* < 0.001), anxiety (*r* = −0.29, *p* < 0.001) and depression (*r* = −0.40, *p* < 0.001), and a significant, positive association with gratitude, school belonging, and grit-P (*r* = 0.25, 0.50, and 0.41 respectively, *p* < 0.001); however, it was not significantly correlated with grit-C (*r* = 0.04, *p* > 0.05). The intercorrelations among all variables are presented in Table 1.

### 3.3. Path Analysis

While controlling for the significant effects of the demographic variables (i.e., age and gender) on mental distress (i.e., depression, anxiety, and stress) and other variables, we first tested the full mediation model, in which grit-C, grit-P, gratitude, and school belonging explained purpose of life, which in turn, accounted for depression, anxiety, and stress. In the model, grit-C, grit-P, gratitude, and school belonging were covariates of each other (based on the results of significant intercorrelation shown in the correlation analyses). The fit indices of this full mediation model were not satisfactory, with χ^2^(18) = 135.94, *p* < 0.001, χ^2^/df = 7.55, CFI = 0.935, SRMR = 0.086, RMSEA = 0.118. Two hypothesized paths were found to be nonsignificant, which were the paths from grit-C and gratitude to purpose in life, suggesting that there were no significant indirect effects from the grit-C and gratitude to mental distress variables. Besides, the modification index tests suggested the addition of direct pathways from grit-C, gratitude, and school belonging to the dependent variables. 

**Figure 1:**
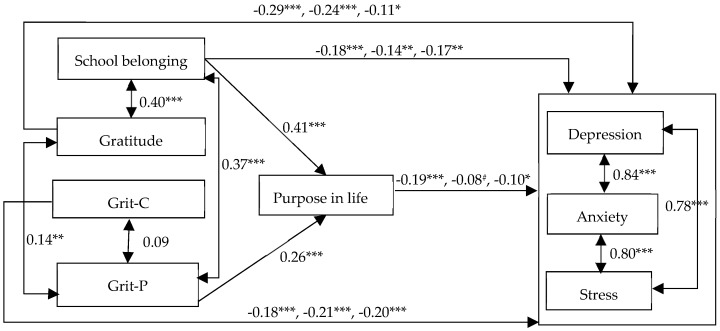
The standardized coefficients of the modified path model. (* *p* < 0.05; ** *p* < 0.01; *** *p* < 0.001; The three figures on those direct paths to mental distress represented the standardized coefficients for the paths to depression (left), anxiety (middle), and stress (right); # The standardized regression coefficients between purpose in life and anxiety was marginally significant (*p* = 0.10). The effect of both gender and age were controlled in the model.)

Given the path analysis results of the full mediation model, we revised the model by dropping the two nonsignificant paths and adding nine direct paths from grit-C, gratitude, and school belonging to depression, anxiety and stress and then tested this modified model. The modified model showed excellent model fit indices, χ^2^(13) = 19.61, *p* = 0.105, χ^2^/df = 1.51, CFI = 0.996, SRMR = 0.025, RMSEA = 0.033. This partial mediation model explained 29.0%, 17.2%, and 13.6% of the variances in depression, anxiety, and stress, respectively (see Figure 1).

In the partial mediation model, the bootstrapping results showed that school belonging had both significant direct and indirect effects on depression with the total effect, −0.25 (95% CI [−0.35, −0.16]) and indirect effect, −0.08 (95% CI [−0.13, −0.04]). It also had significant direct but non-significant indirect effect on anxiety and stress, with the total effects, −0.17 (95% CI [–0.28, −0.06]) and −0.22 (95% CI [−0.32, −0.11]) respectively and indirect effect, −0.03 (95% CI [−0.08, 0.01]) and −0.04 (95% CI [−0.09, 0.001]) respectively. The result suggests that the negative association between school belonging and mental distress (particularly depression) was partially mediated by purpose in life. Gratitude and grit-C had direct, but not indirect, effects on depression (−0.29, 95% CI [−0.38, −0.20] and −0.18, 95% CI [−0.27, −0.10] respectively), anxiety (−0.24, 95% CI [−0.35, −0.14] and −0.21, 95% CI [−0.29, −0.12] respectively) and stress (−0.11, 95% CI [−0.21, −0.01] and −0.20, 95% CI [−0.29, −0.11] respectively). For grit-P, its indirect effect was −0.05 (95% CI [−0.09, −0.02]), −0.02 (95% CI [−0.05, 0.01]) and −0.03 (95% CI [–0.06, 0.01]) on depression, anxiety, and stress respectively. The statistics shows that its effect on mental distress was very weak and even nonsignificant. 

## 4. Discussion

In this study, Chinese emerging adults reported mild or above levels of depression, anxiety and stress symptoms, at the rate of 23.9%, 38.0% and 5.5%, respectively, and these rates were higher than in the general adult population in Macao, which Wu et al. reported to be 19.7% and 26.7%, for depression and anxiety, respectively [59]; such findings suggests that further research attention, to inform prevention and intervention models, should be paid to emerging adults’ mental distress.

The purpose of this study was to examine the extent to which positive psychology factors (i.e., purpose in life, gratitude, grit, and school belonging) serve as protective factors against Chinese emerging adults’ mental distress. As expected, gratitude had a significant negative association with mental distress, especially for depression symptoms. This finding was consistent with previous studies conducted in college students [60,61], which have shown that emerging adults with higher levels of gratitude tend to report less mental distress than those with lower levels of gratitude. Furthermore, consistency of interests, a component of grit, was shown to have a negative relationship with mental distress, whereas perseverance of effort, another component of grit, did not have a significant relationship with it, with the exception of depression. Very limited research has been conducted on testing this relationship, but past findings have shown the relationship between the grit and depression to be mostly negative [62,63]. Our results also supported school belonging as an important protective factor, which was shown to have a significant and negative direct effect on mental distress. Moreover, results suggested that the relationship between school belonging and depression was mediated by purpose in life. In other words, emerging adults who attend school could improve their mental health problems by way of improving their sense of school belonging through purpose in life. 

Based on our findings, positive psychology interventions could be implemented to alleviate emerging adults’ mental distress. Even simple techniques, such as having them list what they feel grateful for and taking action to express their gratitude (i.e., writing letters of gratitude) should be promoted, because these techniques not only foster gratitude but also decrease mental distress and increase positive affect in daily life [64,65]. A recent review showed that gratitude interventions improved subjective wellbeing, while reducing psychopathological symptoms [66]. For example, experimental data demonstrated that gratitude listing (i.e., listing one’s reasons for feeling grateful) improved wellbeing among undergraduate students [67]. A study on university-based psychotherapy services also found that clients achieved greater results when they were provided with a standard psychotherapy plus gratitude writing versus expressive writing [68]. Therefore, gratitude promoting techniques are likely to produce positive results and should be considered in future interventions. 

Another possible intervention is to encourage a sense of school belonging. In-campus student activities to promote school-based support should be organized to help emerging adults improve their sense of school belonging and purpose in life. Previous studies have suggested that fulfilling students’ developmental needs with the help of mentors may also result in increased sense of belonging and purpose among students [47,48]. Freeman, Anderman and Jensen have showed in their study that sense of belonging in college enhances social acceptance, while class-level belonging is associated with motivation and academic self-efficacy [69]. Similarly, programs that target improving school belonging may consider implementation at different levels. For example, faculty level and department level pedagogical caring may be provided.

In this study, consistency of interests had a significant direct effect on all indicators of mental distress, whereas perseverance of effort had a significant, but very weak, effect on depression only. In previous studies on positive mental outcomes such as life satisfaction, perseverance of effort was a significant and even stronger factor (than consistency of interest) among not only Filipino undergraduate students [31,70] but also general populations [36]. However, our findings suggest that, compared to consistency of interest, perseverance of effort may be a less salient factor for protecting Chinese emerging adults from negative mental outcomes (e.g., mental distress). Further research is warranted to test whether such differential effects of the two grit components on mental distress are consistent in other age and cultural groups. Moreover, given the limited research on how to promote grit, future studies should also explore methods or techniques to improve grit, especially with respect to consistency of interests.

Despite the significant effect on mental distress, consistency of interest had no significant positive association with purpose in life, gratitude, or school belonging in this study. These findings echo a previous study in which, compared with consistency of interests, perseverance of effort was found to be a stronger predictor for positive outcomes (e.g., academic adjustment, college satisfaction, sense of belonging) [31,71]. Datu et al. argued that such differential effects of the two dimensions of grit could be explained by cultural context [31]. It has been proposed that, because the Chinese culture has a collectivist orientation, Chinese tend to value relationship harmony, are more context-sensitive, and they do not necessarily endorse a consistent interest in long-term personal goals; they also possess more a dialectical mindset that allows for more tolerance of contradictions [72]; this may result in consistency of interests being a less important predictor than perseverance of effort for positive psychosocial outcomes [31].

Several limitations exist in the present study. First, use of a convenience sample of university students tends to limit the generalizability of the findings to all Chinese emerging adults. Second, due to the cross-sectional study design, we cannot make causal inferences; in other words, the findings cannot explain the causal relationship between positive psychology variables and mental distress. Third, the study used a self-report tool to measure mental distress, and self-report instrument may lack the diagnostic validity, and the factors of DASS only measure three aspects of mental distress, namely, depression, anxiety, and stress. Further longitudinal study is recommended to investigate the causal relationship between these positive psychology variables and mental distress assessed by clinical diagnostic tools.

## 5. Conclusions

Our findings suggest that school belonging and perseverance of effort has both a direct and indirect protective impact on mental distress, and their indirect impact is mediated by purpose in life. Positive psychology factors, such as gratitude and consistency of interest have direct, protective effects against mental distress among Chinese emerging adults. The two components of grit may have differential effects on mental distress, and further research is warranted to test the replicability of the findings in other age and cultural groups. According to the findings, interventions with positive psychology methods and school-based support is recommended for students in their emerging adulthood.

## Figures and Tables

**Table 1:** Descriptive statistics and correlations of all the variables.

Variables	Mean	SD	1	2	3	4	5	6	7	8
1. Depression	5.91	4.92	1.00	-	-	-	-	-	-	-
2. Anxiety	6.37	4.76	0.86 ***	1.00	-	-	-	-	-	-
3. Stress	7.37	4.58	0.80 ***	0.83 ***	1.00	-	-	-	-	-
4. Grit-C	2.53	0.63	−0.21 ***	−0.23 ***	−0.22 ***	1.00	-	-	-	-
5. Grit-P	3.49	0.61	−0.15 **	−0.03	−0.07	0.09 *	1.00	-	-	-
6. Gratitude	28.66	5.14	−0.42 ***	−0.32 ***	−0.22 ***	0.04	0.15 **	1.00	-	-
7. School belonging	3.97	0.58	−0.40 ***	−0.29 ***	−0.28 ***	0.05	0.39 ***	0.40 ***	1.00	-
8. Purpose in life	19.85	4.11	−0.36 ***	−0.22 ***	−0.23 ***	0.04	0.41 ***	0.25 ***	0.50 ***	1.00

Note: Grit-C represented the consistency of interests and Grit-P represented the perseverance of effort. * *p* < 0.05; ** *p* < 0.01; *** *p* < 0.001.
